# Use of streamnormal forces within an array of tidal power harvesters

**DOI:** 10.1371/journal.pone.0270578

**Published:** 2022-07-06

**Authors:** Ignazio Maria Viola, Zhi Gao, James Smith

**Affiliations:** School of Engineering, Institute for Energy Systems, University of Edinburgh, Edinburgh, United Kingdom; Huazhong University of Science and Technology, CHINA

## Abstract

Tidal energy is a renewable and promising energy source. Turbines are deployed under water in marine channels where there is a fast tidal current. The first arrays have only recently been deployed and there is no consensus yet on the optimal array design. In this paper, we explore whether the maximum harvestable power can be increased by using harvesters or flow deflectors that exert a side force in the streamnormal direction to oppose the expansion of the streamtube. The power harvesting and the side force exertion are modelled with sink terms in the two-dimensional Reynolds-averaged Navier-Stokes equations. We found that the power limit increases linearly with the side force, and it should be exerted from the upstream side edges of the array. We conclude that future arrays might not be made only of turbines that harvest power, but also of deflectors such as vertical wings of size comparable to a turbine. The promising results of this theoretical study may direct new research on the use of deflectors to maximise the power harvested by tidal arrays.

## 1 Introduction

Tidal energy is a renewable and predictable energy source expected to contribute more than 70 GW to the global energy production by 2050 [1]. Given the first full-scale tidal energy array, Maygen, has only just been deployed in the UK, there remains much scope to further the theory and understanding behind this renewable energy generation method. The constraint imposed by the free surface on the flow stream makes the fluid mechanics of tidal energy extraction profoundly different from, for instance, that of wind energy. Recent reviews on the fluid mechanics modelling of tidal power harvesters and arrays are given by Adcock et al. [2].

The maximum power that can be extracted from a uniform flow stream has been investigated by Lanchester [3], Betz [4] and Joukowsky [5]. They independently found the upper extraction limit to be 16/27 of the undisturbed kinetic energy flux through an area equal to that of the harvester. Garret and Cummins [6] revealed that this limit can be overcome when the stream is constrained in a two-dimensional channel such as a shallow tidal channel. Specifically, the maximum power increases by a factor of (1 − *B*)^−2^, where *B* = *A*/*A*_*c*_ is the fraction of the channel area *A*_*c*_ occupied by harvester area *A*, referred to as the blockage ratio. This result was later demonstrated algebraically by Dehtyriov et al. [7].

Nishino and Wilden [8, 9] showed that these same underlying blockage mechanisms also occur as a result of the local blockage between neighbouring turbines. The implication of this finding is that harvesters should be deployed side by side in a row [10]. In-line closely packed harvesters is a unique optimal layout to power harvester arrays where the flow is constrained in one plane.

Because of the different length scales for the flow around the harvesters and the array, the maximum power depends on both the local blockage due to the neighbouring turbines, and the global blockage due to the fraction of the channel area occupied by the array [11]. The separation of scales was initially considered by Nishino and Wilden [8], and then further extended to three scales (arrays of arrays) by Cooke et al. [12] and eventually to infinite scales with a fractal approach by Dehtyriov et al. [7].

These above-mentioned theoretical studies suggest that the maximum power extraction is achieved for a fence-like one-line array, which spans across the entire channel width. This result is relevant to tidal energy. However, such arrays would result in a substantial impact on navigation and the environment. Consequently, it is likely that highly dense tidal power arrays will only be deemed acceptable if they extend over a small fraction of the tidal channel. In this case, the global blockage is negligible and the array can be modelled as in an infinitely wide channel.

In the case of tidal energy, the flow is not entirely constrained because the sea surface is free to deform. The effect of the free surface deformation resulting from the power extraction was considered by, for instance, Whelan et al. [13], Vogel et al. [14], and Gupta and Young [15]. This effect is significant only at high Froude numbers, and is likely to be negligible in most of the practical applications.

All of the above studies consider only the streamwise force exerted by the harvesters on the fluid. However, harvesters might also exert a force in the streamnormal direction. This is the effect is observed in the case of a yawed turbine, for example. The ad-hoc use of these forces might result in a higher energy extraction, and this has never been investigated before.

This paper sets out to explore whether it is possible to enhance the harvested power by exerting a side force in the streamnormal direction. The underlying principle is akin to the radial force exerted by the duct of a constrained turbine or a propeller. Pioneering work on ducted rotors was undertaken by Betz [16]. As shown by Conway [17], and more recently by Bontempo and Manna [18], the coupling of the actuator disk theory with a vortex system that accounts for the duct and the wake is not trivial. Hence, the coupled problem is often investigated numerically or experimentally. A review of the theoretical models is presented by Bontempo and Manna [19], while Nunes et al. [20] reviewed the progress towards the optimal design.

While the effect of applying a side force around a turbine is well understood, the application of a side force distributed ad-hoc throughout the array has never been investigated. Specifically, the novelty of this paper is to address the following questions: (1) how does the harvested power vary when exerting a side force distributed over the array? (2) Which devices within the array should exert the side force? (3) Should devices be used to simultaneously harvest power and exert a side force?

The rest of the paper is organised as follows. In §2, we explore the theoretical principles upon which our proposal is built. §3 details the numerical method, including design of the computational domain and key results from the uncertainty calculations. Results are presented and discussed for a uniform distribution of side force through the array in §4, and for the side force exerted by deflectors placed at the edges of the array in §5. A discussion of the results including the implications on the optimal design is presented in §6. Conclusions are drawn in §7.

## 2 Theoretical considerations

**Fig 1:**
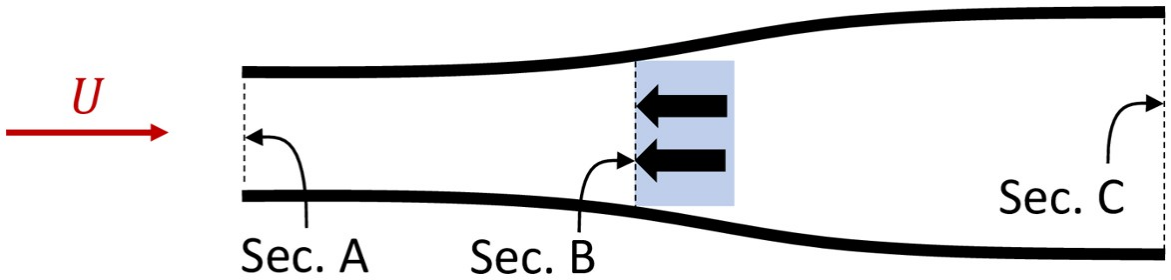
Time averaged streamlines for two conventional power harvesters without side force. The red arrow shows the tidal stream current velocity, black arrows indicate the forces exerted on the flow by the harvesters (in blue), black lines represent streamlines.

Firstly, consider conventional power harvesters that *do not* exert a side force. Fig 1 shows the streamlines around two harvesters placed side by side. The flow through the streamlines is the streamtube from which power is harvested. Each black arrow represents the force exerted on the flow as a result of the power extraction. This force is equal in magnitude and opposite in direction to that experienced by the harvester, known as thrust. Flow cannot cross the streamlines, hence the mass flow rate across sections A and C is conserved. Because the cross sectional area increases from A to C, continuity of mass flow rate dictates the flow velocity decreases from upstream to downstream of the harvester.

**Fig 2:**
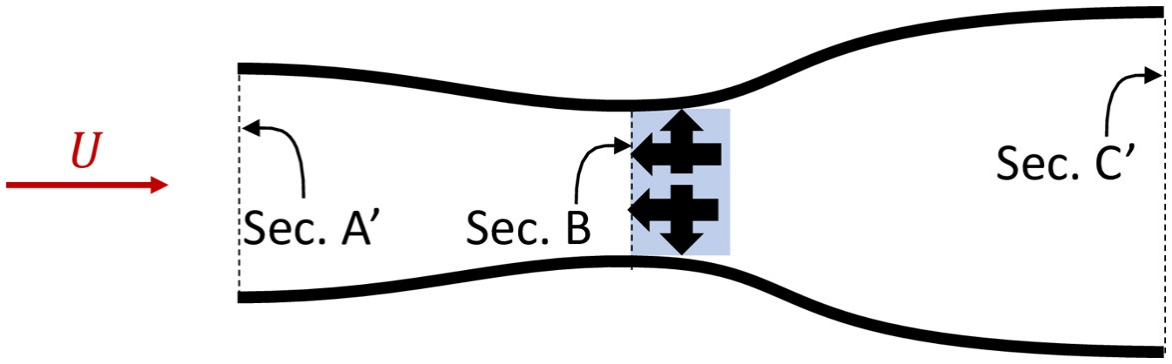
Time averaged streamlines for two power harvesters with side force exertion. The red arrow shows the tidal stream current velocity, black arrows indicate the forces exerted on the flow by the harvesters (in blue), black lines represent streamlines.

Now consider two power harvesters at B exerting side forces orthogonal to the flow in opposite directions (Fig 2). The effect of these side forces is to curve the streamlines inward between A’ and B, effectively increasing the area of the streamtube from which the power is extracted (area A’), for the same area occupied by the turbines (area B).

**Fig 3:**
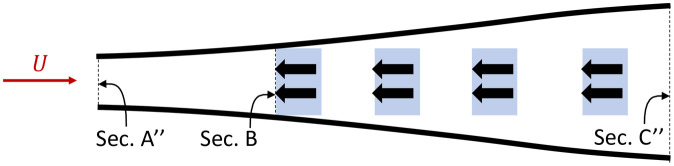
Time averaged streamlines for a conventional array of power harvesters without side force exertion. The red arrow shows the tidal stream current velocity, black arrows indicate the forces exerted on the flow by the harvesters (in blue), black lines represent streamlines (to scale).

Fig 3 shows the diverging streamlines calculated for an array of conventional harvesters. For illustrative purposes we use four rows of two parallel harvesters, but the concept may be extended to any number of rows and harvesters. It is observed that the flow velocity decreases for each subsequent downstream harvester, thus each extracts less power than the one before. In fact, having beyond 5-6 rows of turbines can significantly reduce the overall *C*_*p*_ of the tidal array [21, 22], which could render the farm economically unviable.

**Fig 4:**
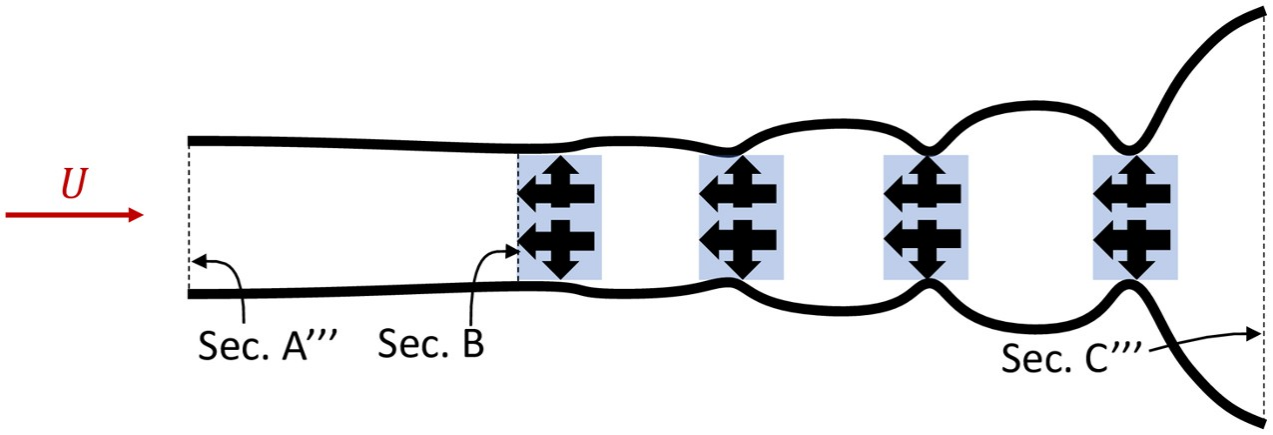
Time averaged streamlines for an array of power harvesters with side force exertion. The red arrow shows the tidal stream current velocity, black arrows indicate the forces exerted on the flow by the harvesters (in blue), black lines represent streamlines (to scale).

Fig 4 shows similar calculations but with an optimised side force delivered by each harvester. These side forces keep the velocity constant within the array and thus increase the amount of power extracted. As illustrated, area A”’>>A” and C”’>>C” which demonstrates a higher flowrate through the array and a larger wake downstream of the array, i.e. greater power extraction.

In order to exert a side force, the harvester might use some power depending on the technology and kinematics employed. However, in principle, a side force can be exerted without the use of any power. This is analogous to the application of ducts, which have been shown to provide multiple-fold increases in turbine power output by shaping the streamlines around the turbine [23]. Here, we propose using a side force to achieve a similar effect. The side force generated by the harvesters in Fig 4 is equivalent to four ducts in series, with diameters of the scale of the array width.

## 3 Method

To model the harvesters, we consider only the total force that they exert on the fluid. Because we consider a flow constrained in one dimension, such as the constraint due to the shallow water condition of the tidal channel, we make a two-dimensional approximation, where the velocity and forces are averaged over the constrained direction. We therefore model the power extracted by the harvester by considering the force density *F*_*x*_ (N m^−3^) exerted on the fluid in the harvester region. The underlying assumption of this two-dimensional model is that the combined force of the harvester(s) is distributed across the harvester region. This method is the approach taken by, for example, Nishino et al. to model wind turbine [24] and tidal turbine [9] arrays. It is noted that this approach is almost scale independent. In fact, each volume within which the flow and force is considered uniform can represent either a small volume with only one harvester (e.g. a turbine), or an entire array of harvesters.

Eq 1 defines the power extracted per unit depth within the harvester region,

$$\begin{array}{c} P=F_{x}WLU, \end{array}$$ (1)

where *P* is the power extracted from the flow and *U* is the average flow velocity within the harvester region. The energy flux per unit depth available in the undisturbed flow is $\rho WU_{0}^{3}/2$, where *ρ* is the fluid density and *U*_0_ is the freestream velocity in the unexploited stream. The power coefficient is the ratio between the power extracted and the energy flux per unit depth of the unexploited stream,

$$\begin{array}{c} C_{P}=\frac{F_{x}WLU}{\frac{1}{2}\rho WU_{0}^{3}}=\frac{F_{x}LU}{\frac{1}{2}\rho U_{0}^{3}}. \end{array}$$ (2)

For the purposes of this study, it is important to distinguish between the unexploited energy flux, *P*_0_, defined by *U*_0_, and the exploited energy flux, *P*, defined by *U*. Both fluxes are defined per unit depth based on the two-dimensional assumption. We therefore define a local power coefficient based on the velocity *U* as

$$\begin{array}{c} C_{P}^{\prime }=\frac{F_{x}WLU}{\frac{1}{2}\rho WU^{3}}=\frac{F_{x}L}{\frac{1}{2}\rho U^{2}}. \end{array}$$ (3)

Lanchester, Betz and Joukowsky [3–5] showed that there exists an optimal harvester force density *F*_*x*,opt_ for which *C*_*P*_ is maximised, and thus a corresponding optimal $C_{P}^{\prime }$ exists such that:

$$\begin{array}{c} F_{x,\text{opt}}=\frac{\frac{1}{2}\rho U^{2}}{L}C_{P,\text{opt}}^{\prime }. \end{array}$$ (4)

We also consider harvesters that exert a side force orthogonal to the freestream. We call these harvesters *deflectors*, whose side force per unit volume is *F*_*y*_ (N m^−3^). We define the side force coefficient as

$$\begin{array}{c} C_{y}=\frac{F_{y}\;L}{\frac{1}{2}\rho V^{2}}, \end{array}$$ (5)

where *V* is the average sidewise (streamnormal) velocity within the harvester region.

### 3.1 Deflector types

For the purposes of this study, we focus our attention to three deflector concepts. Each are characterised by the stream-opposing force density ($F_{x_{1}},F_{x_{2}},F_{x_{3}}$) they impart on the flow in addition to side force density *F*_*y*_. They are as follows:

1. Power-extracting deflector:
  $$\begin{array}{c} F_{x_{1}}=\frac{\frac{1}{2}\rho U^{2}}{L}C_{P,\text{opt}}^{\prime }. \end{array}$$ (6)
2. Zero-thrust deflector:
  $$\begin{array}{c} F_{x_{2}}=0. \end{array}$$ (7)
3. Power-neutral deflector:
  $$\begin{array}{c} F_{x_{3}}=\frac{F_{y}V}{U}. \end{array}$$ (8)

Here, *U* and *V* refer to the average streamwise and sidewise velocity values over the deflector region respectively. Although these deflector concepts in their optimal forms exist only in theory, one might compare their function to the following real phenomena. Deflector 1 acts to partially deflect the flow, while extracting as much of the remaining streamwise power as possible. This has similarities with, for example, a yawed turbine whose axis is not aligned with the free stream. Deflector 2 directs the flow sideways without providing any flow resistance. This result is akin to that provided by a plasma actuator in aerodynamic applications. Deflector 3 is akin to a curved vane, changing the direction of the flow from *U* to *V*.

### 3.2 Computational setup

While the side force can be modelled in potential flow through the application of inviscid lift-generating vortices, the streamwise force would require a more complex utilisation of sources and sinks, or an actuator disk approach [18]. Instead, we solve the Navier-Stokes equations with a finite volume approach, which allows for any arbitrary set of forces to be exerted on any fluid region. Specifically, we solve the steady, incompressible, two-dimensional Reynolds-averaged Navier-Stokes equations for a Newtonian fluid, that are

$$\begin{array}{c} \frac{\partial u}{\partial x}+\frac{\partial v}{\partial y}=0, \end{array}$$ (9)

$$\begin{array}{c} u\frac{\partial u}{\partial x}+v\frac{\partial u}{\partial y}=\frac{1}{\rho }\frac{\partial p}{\partial x}+\left(\nu +\nu_{t}\right)(\frac{\partial^{2}u}{\partial x^{2}}+\frac{\partial^{2}u}{\partial x\partial y}+\frac{\partial^{2}u}{\partial y^{2}})+\frac{F_{x}}{\rho }, \end{array}$$ (10)

$$\begin{array}{c} u\frac{\partial v}{\partial x}+v\frac{\partial v}{\partial y}=\frac{1}{\rho }\frac{\partial p}{\partial y}+\left(\nu +\nu_{t}\right)(\frac{\partial^{2}v}{\partial x^{2}}+\frac{\partial^{2}v}{\partial x\partial y}+\frac{\partial^{2}v}{\partial y^{2}})+\frac{F_{y}}{\rho }, \end{array}$$ (11)

where *u* and *v* are the flow velocities in the *x* (streamwise) and *y* (streamnormal) directions respectively, *ν* and *ν*_*t*_ are the kinematic viscosity and the eddy turbulent viscosity, respectively. The latter is modelled with the Spalart-Allmaras turbulence model. We use the code STAR CCM+ 13.04, with a second order upwind numerical scheme.

**Fig 5:**
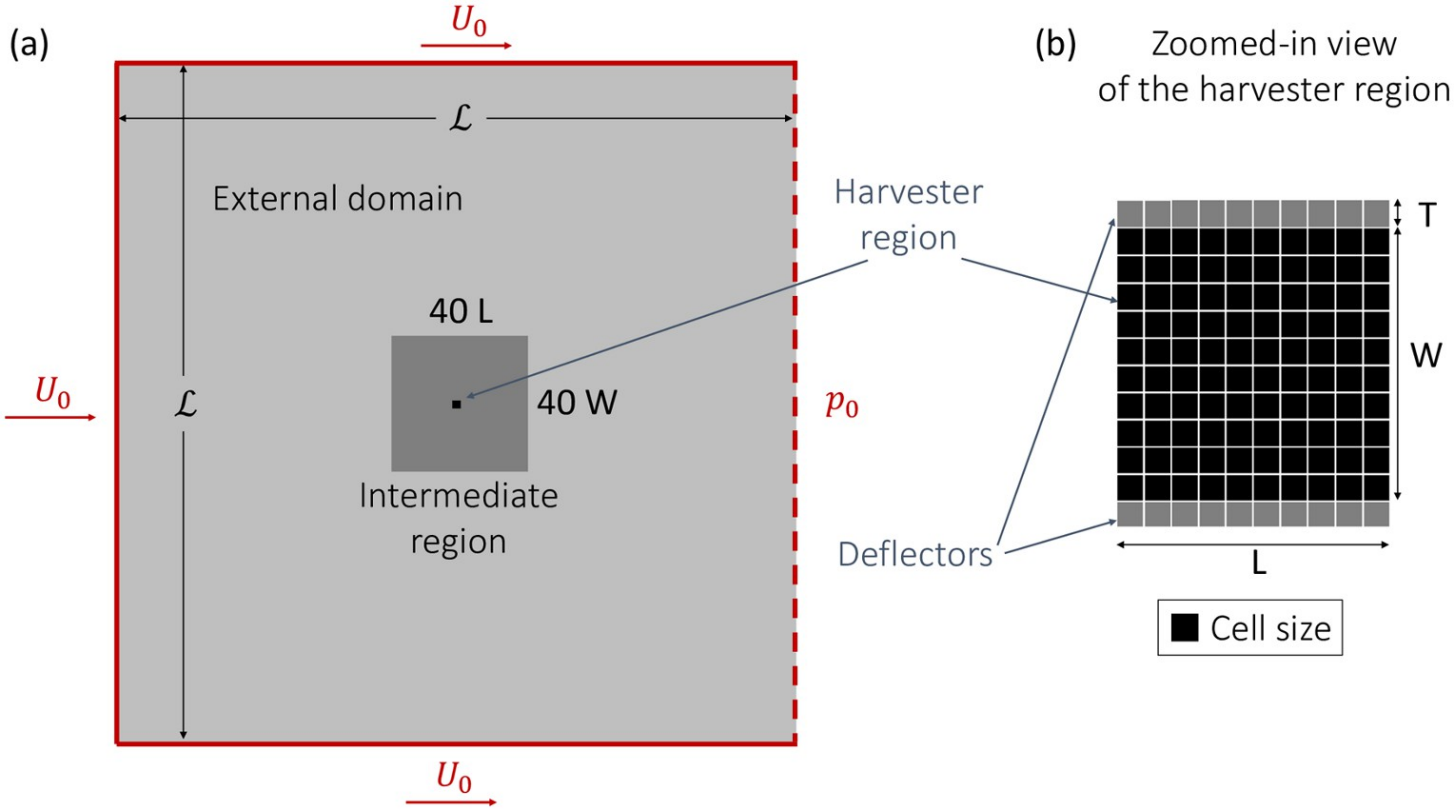
Overview of the computational domain (a) and detail of the harvester region (b). The boundary conditions applied to the external edges of the domain are shown in red. Dark grey in (a) shows the inner region with the first level of grid refinement, and and the black region shows the harvester region. Dark grey in (b) shows the layers of deflectors around the harvesting region that will be considered in §5.

Fig 5 depicts the computational domain and the boundary conditions. The external domain is a square area with length L=100L, where a uniform constant velocity *U*_0_ and *ν*/*ν*_*t*_ = 10 is set on the upstream and side boundaries, while a constant pressure *p*_0_ is set on the downstream boundary. The non-conformal grid made of squared elements is refined in an intermediate region 40*L* long and 40*W* wide, and then again around the harvester region (*L* × *W*). For those conditions where the side forces are symmetrically exerted, only half of the domain with a symmetry boundary condition is considered.

**Fig 6:**
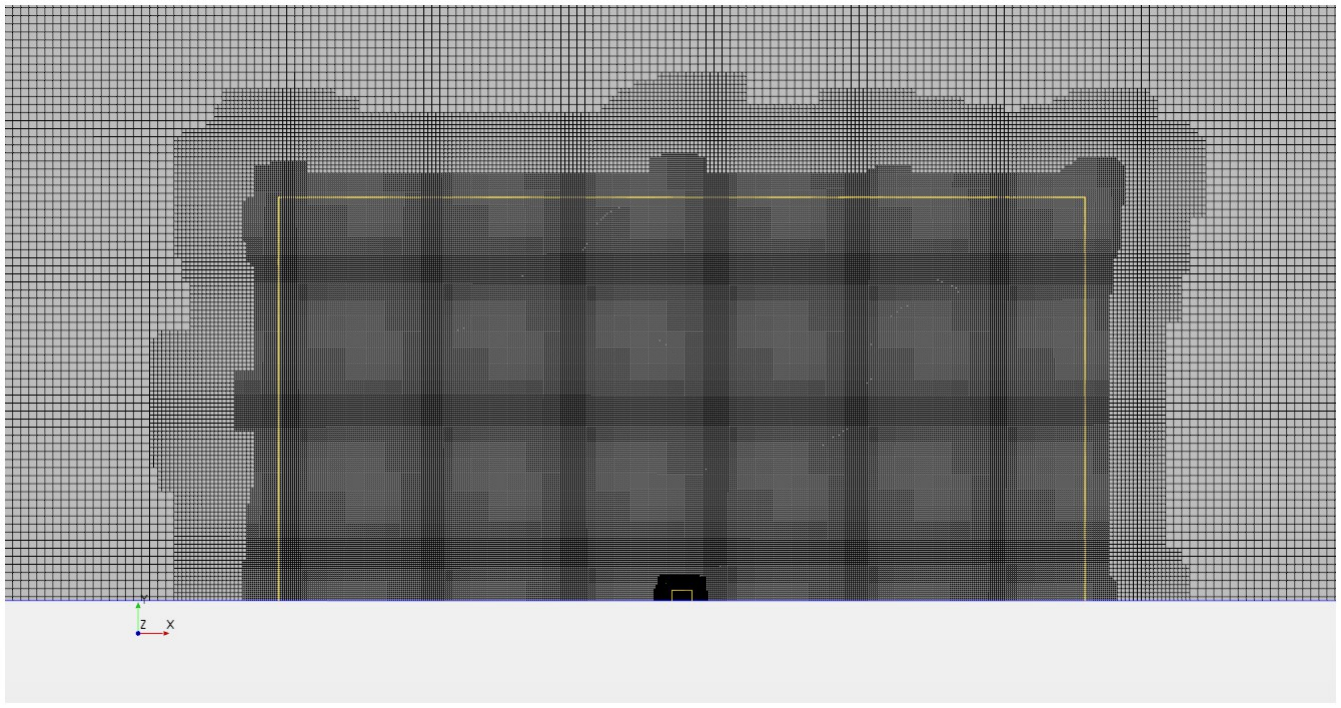
Mesh of half of the computational domain (top half with respect to Fig 5a). Not all of the exterior region is shown. The yellow line marks the boundary of the intermediate region.

In §4, we will consider a uniform array represented by the harvester region, where the harvesters are also deflectors. Conversely, in §5, we will apply side force from the array edges only by placing deflectors on the two sides of the array within regions of thickness *T* (as shown in Fig 5b). Fig 6 depicts half of the meshed domain, where the flow direction is from left to right. The edge length of each grid cell is *W*/20 in the harvester region, *W*/10 in the intermediate region, and 2*W*/5 in the external region.

We set the Reynolds number *Re* = *U*_0_ *W*/*ν* to 10^7^. It will be shown in §4.2 that the results are not sensitive to *Re*. Nevertheless, the *Re* is intentionally selected as the minimum realistic value to assess any viscous effects at their most significant. To deliver this lower limiting condition, we define a flow stream with velocity *U* = 1 m s^−1^, and a harvester region with a width *W* = 10 m. For these conditions, the grid resolution corresponds to 500 mm. We expect that the results would remain valid for higher flow velocities and array dimensions.

### 3.3 Model verification

The uncertainty associated with the grid resolution is assessed following the method proposed by Viola et al. [25]. We consider $F_{x}=F_{x_{1}}$ and *F*_*y*_ = 0 in the harvester region, while *F*_*x*_ = *F*_*y*_ = 0 elsewhere. We assess the maximum power coefficient for six different grid resolutions. The finest and the coarsest grids have a linear grid size (Δ*x*) that is 0.5 and 1.4, respectively, of the size Δ*x*_base_ of the base grid (shown in Fig 6).

**Fig 7:**
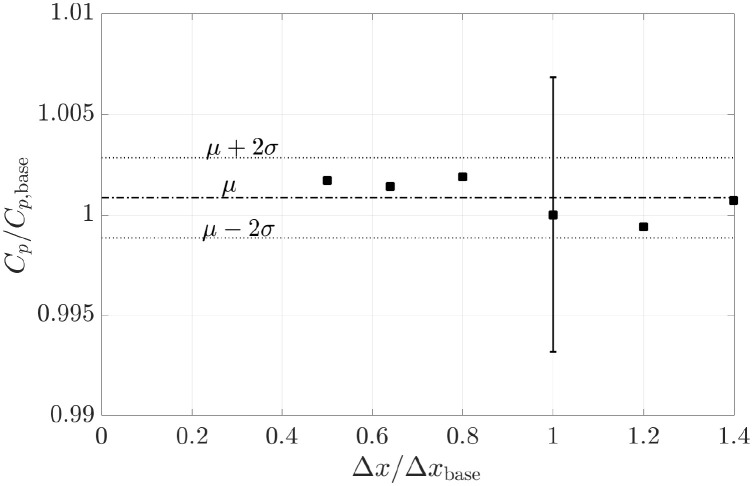
Relative variation of the maximum power coefficient versus the relative linear grid size. The mean value *μ* and the band within two standard deviations *σ* of the set of *C*_*p*_/*C*_*p*,base_ values are shown with a dash-dot line and dashed lines, respectively. The vertical bar shows the uncertainty at 95% confidence interval in the estimate of *C*_*p*_ with the base grid.

The power coefficient is found to vary by less than 0.3% for all tested grids (Fig 7). Because the results do not show a clear trend, the uncertainty at 95% confidence interval is estimated as

$$\begin{array}{c} U_{C_{p}}=1.5(\text{max}\frac{C_{p}}{C_{p,\text{base}}}-\text{min}\frac{C_{p}}{C_{p,\text{base}}})=1.4\%. \end{array}$$ (12)

While results for $F_{x}=F_{x_{2}}$ and $F_{x_{3}}$ can be computed explicitly, simulations with $F_{x}=F_{x_{1}}$ require an iterative approach comparing a range of simulations to determine *F*_*x*,opt_. We use an extensive search by varying $C_{P}^{\prime }$ in steps of 0.2 and we set $C_{P,\text{opt}}^{\prime }$ as the value corresponding to the maximum *C*_*p*_. Around the optimal value, a step of 0.2 in $C_{P}^{\prime }$ results in maximum error in *C*_*p*_ of 1%.

## 4 Uniform force distribution within the harvester region

In this section we consider forces that are uniformly distributed throughout the harvester region. First, the effect of only a streamwise force exerted by the whole harvester region is considered (§4.1). This allows the comparison of the results with the Lanchester-Betz-Joukowsky’s law, and the assessment of the effect of the two geometric parameters of the model: the domain size L and the harvester region length *L*. Subsequently, in §4.2, the effect of both a streamwise and a side force exerted from the whole harvester region is considered.

### 4.1 Power harvesting

Consider a harvester region without side force, where $F_{x}=F_{x_{1}}$ and *F*_*y*_ = 0. For the base geometry and grid resolution (§3.3), the maximum power coefficient is *C*_*p*_ = 0.639. We would expect a similar power coefficient to that predicted by the Lanchester-Betz-Joukowsky’s law, that is *C*_*p*_ = 0.593. Instead, the simulation predicts a 7.7% higher *C*_*p*_. Beyond the small error (±0.7%), the discrepancy could be due to the effect of the finite domain size, and the entrainment of momentum over the length *L* of the harvester region. These effects are hereby investigated.

**Fig 8:**
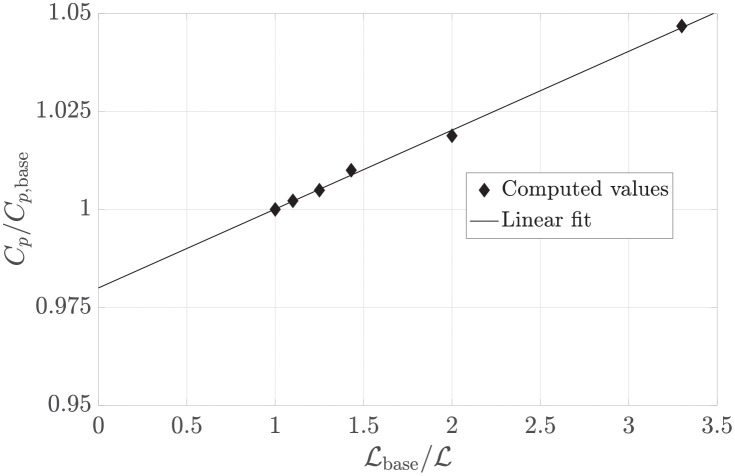
Relative variation of the maximum power coefficient versus the relative linear domain size. The intercept of the linear fit with the ordinate shows the expected *C*_*p*_/*C*_*p*,base_ for an infinite domain size (L→∞).

In inviscid flow, the effect of the harvester extends to infinity. Therefore, velocity and pressure would not be uniform where the external domain boundaries are located. To test the effect of imposing a uniform velocity and pressure at a finite distance from the harvester region, the domain dimension L is decreased in steps up to 0.3 of the base value ($L_{base}$). Fig 8 shows that the power coefficient decreases with the inverse of the domain size. By extrapolating for an infinite domain ($L_{\text{base}}/L=0$), the power coefficient would decrease by about 2% (*C*_*p*_/*C*_*p*,base_ = 0.98).

**Fig 9:**
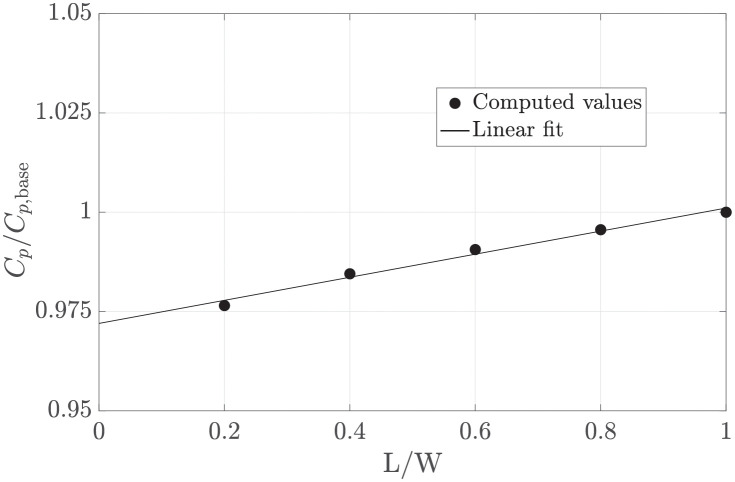
Relative variation of the maximum power coefficient versus the aspect ratio of the harvester region. The intercept of the linear fit with the ordinate shows the expected *C*_*p*_/*C*_*p*,base_ for a vanishing length (*L* → 0).

The harvester region is taken as squared, with *L* = *W*. To demonstrate that the aspect ratio *L*/*W* has a marginal effect on the conclusions, and the extent to which this contributes to the observed differences with the Lanchester-Betz-Joukowsky’s law, we compute the maximum power coefficient for harvester regions with a length ranging from *L* = 0.2*W* to *L* = *W*. We expect a marginal power increase with *L* due to the additional momentum that entrains through the side boundaries of the harvester region. This is shown in Fig 9. For *L* → 0, the maximum power coefficient would decrease by about 3% compared to the base case with *L* = *W*.

### 4.2 Side force

**Fig 10:**
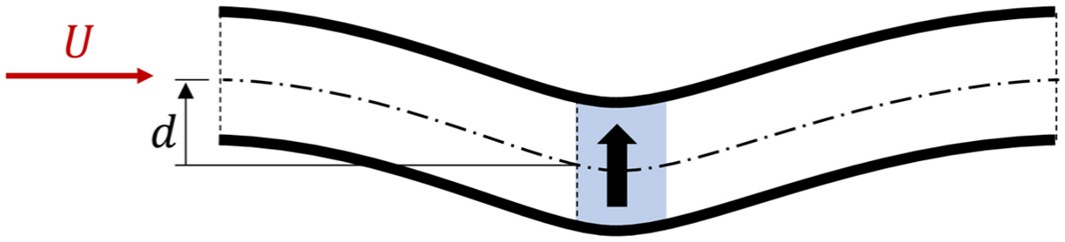
Streamlines around a deflector. The black arrow indicates the force exerted on the flow by the deflector (in blue), dash-dot line shows the axis of the streamtube, and *d* is its displacement due to the side force.

In this section we investigate how the side force deflects the streamtube through the array. If there was only a thrust force, we would expect an expansion of the streamtube through the harvester, but the axis of the streamtube would remain straight through the axis of the harvester. Conversely, if we only had a side force, we would expect the axis of the streamtube to be deflected. For example, in Fig 10, under application of side force, *F*_*y*_, the axis of the streamtube is deflected in the direction of *F*_*y*_. To ensure conservation of mass, the axis of the streamtube is deflected upwards both upstream and downstream of the deflector.

The deflection of the streamtube axis can also be computed with potential flow theory. The deflector can be substituted for a point vortex with circulation

$$\begin{array}{c} \Gamma =\frac{F_{y}WL}{\rho U}. \end{array}$$ (13)

**Fig 11:**
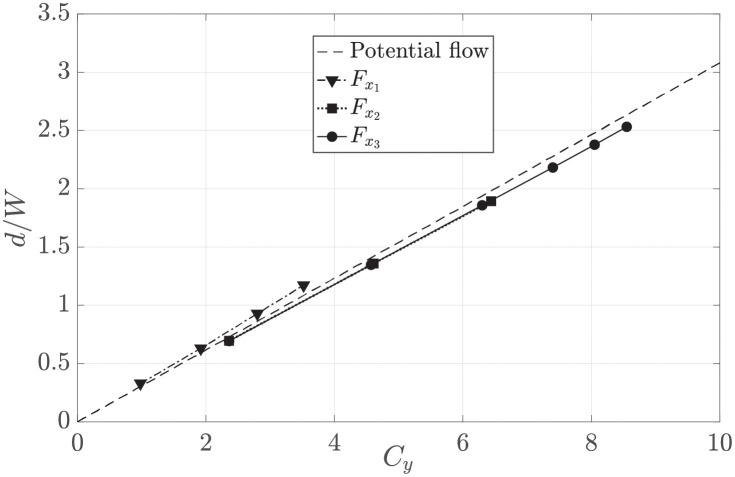
Displacement of the streamtube axis versus the side force coefficient for the three deflectors types, and as predicted by potential flow theory.

Fig 11 shows the results from the simulations against the potential flow solution, where the displacement *d* of the axis streamtube is measured at the domain upstream edge (50*L* upstream of the centre of the harvester region). The relationship between side force magnitude and streamline deflection is clearly illustrated to be linear in all deflector types, and shows good agreement with the potential flow solution. For the same side force, all three deflector types deflect the streamtube by an equal magnitude. Therefore, the variation of thrust force *F*_*x*_ and, thus, magnitude of power extracted has little effect on the deflection of the streamtube axis.

The agreement between the Navier-Stokes solution and potential flow theory suggests that the solution is effectively Reynolds number independent. In the Navier-Stokes solutions, vorticity is generated through the harvester region because a streamwise force is exerted [26]. If the Reynolds number is sufficiently high, the effect of the Reynolds number is only a marginal increase in the wake diffusion. This effect can be quantified considering how *C*_*p*_ varies with the length of the harvester region (Fig 9). We have previously showed (§4.1) that the entrainment of momentum along *L* only contributes to 3% of the harvested power.

### 4.3 Power harvesting with side force exertion

In this section we consider how the maximum harvested power varies by exerting a side force. Specifically, we aim to address the following question: If a side force is exerted over a region, is the maximum power harvested within the region going to be affected? To investigate, we compute the power harvested by the the deflector that aims at maximising the harvested power ($F_{x_{1}}$) for different values of the side force.

**Fig 12:**
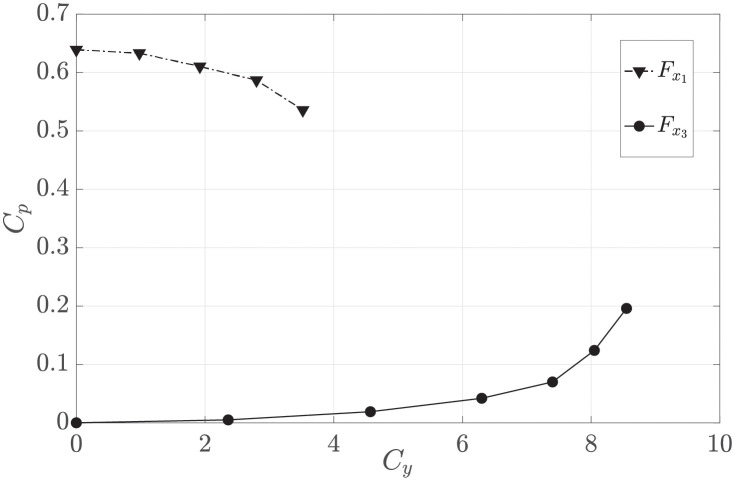
Power coefficient versus the side force coefficient for the two types of deflectors that harvest power. Deflectors are placed within the harvester region (see Fig 5).

Fig 12 shows that *C*_*p*_ varies marginally for small *C*_*y*_, while it decreases at a fast rate for high *C*_*y*_. This trend is due to the following. Assume for simplicity that the side force is exerted along a line parallel to the freestream velocity. The line splits the harvester region in two parts, one where the flow is accelerated by the side force and where more power can be harvested, and one where the flow is decelerated. Overall the gain in power harvested is negligible. This shows that the deflectors should be placed on the side edges of the array to maximise their efficacy.

For high *C*_*y*_, in most of the harvester region a substantial part of the streamwise velocity is redirected to the streamnormal direction, and thus *C*_*p*_ decreases. This is also evident in the *C*_*p*_ trend of the deflector $F_{x_{3}}$, which harvests only the power needed to exert the side force (Fig 12). The deflector does not harvest any power for small *F*_*y*_, but harvests increasing quantities of power as the streamnormal velocity component increases at high *C*_*y*_.

## 5 Side force at the array edges

In this section, we investigate the power increase that can be gained by placing the deflectors at the sides of the array. Henceforth, we consider only deflectors of the third type, $F_{x_{3}}$, while the maximum power is harvested in the harvester region. First we consider a uniform distribution of deflectors along the two sides, and successively we investigate the benefit of non-uniform distributions.

**Fig 13:**
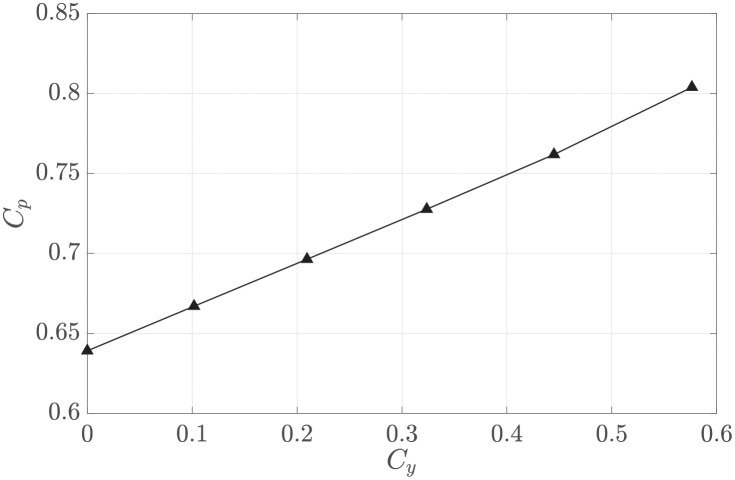
Power coefficient versus the side force coefficient for the deflector type $F_{x_{3}}$ placed within a region at the side edges of the harvester region.

Consider a side force uniformly distributed along over two regions, length *L* and width *T*, at the side edges of the array (Fig 5). Fig 13 shows how the array power coefficient *C*_*p*_ performs with increasing side force. Within the range of *C*_*y*_ tested, *C*_*p*_ increases linearly with *C*_*y*_ by more than 25%.

**Fig 14:**
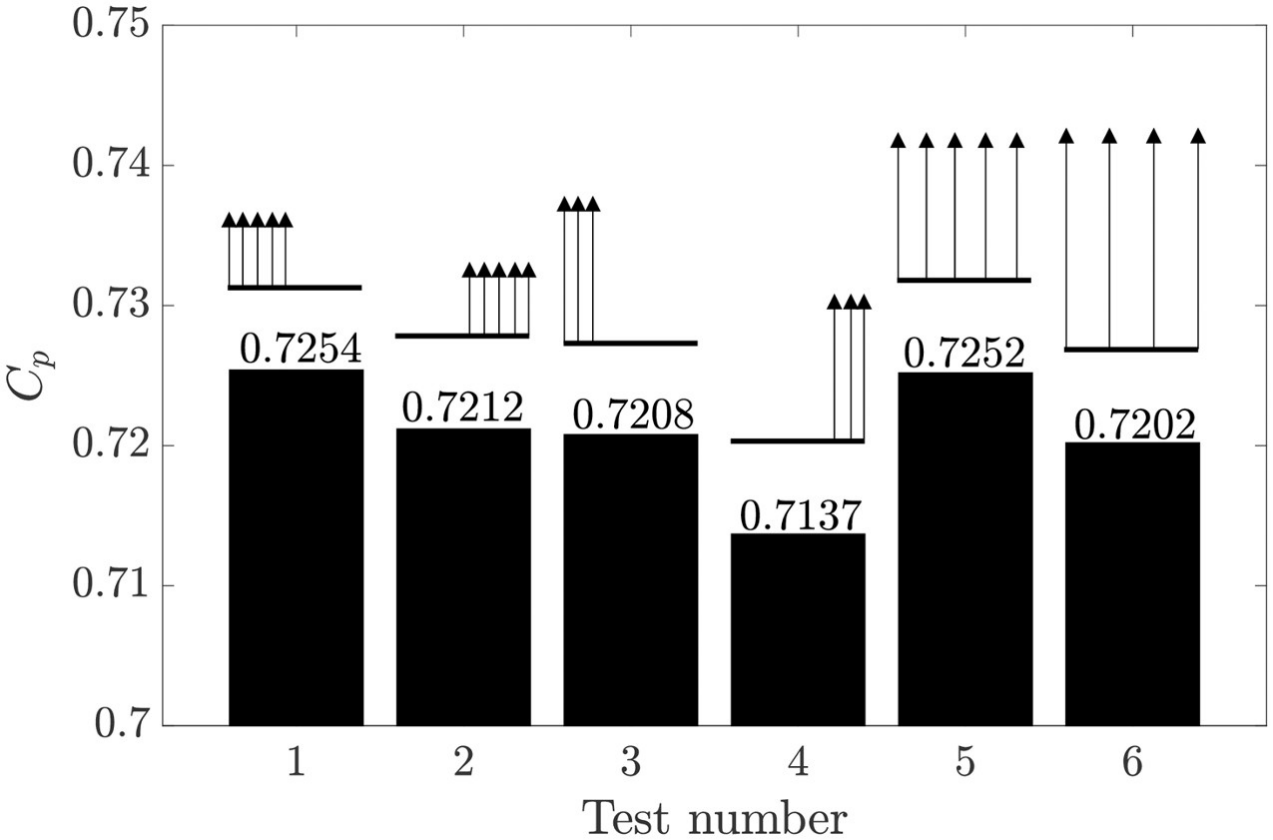
Power coefficient for six different streamwise distributions of the side force exerted by deflector type $F_{x_{3}}$ at the side edges of the harvester region. The distributions of the side force along the length *L* of the deflector region is shown by the ten arrows, whose length is proportional to the side force exerted.

We now turn our attention to the streamwise distribution of the side force. Fig 14 shows maximum *C*_*p*_ in the harvester region for several discretised side force delivery configurations. As shown in Fig 5, the perimeter regions are divided into ten separate deflector regions, each exerting side force. The arrows in the diagrams above each test case in Fig 14 indicate which of the ten deflectors are exerting side force, left being upstream and right being downstream. In each case, the total side force delivered is the same.

It can be seen that cases in which a portion of the force is delivered at the upstream side of the harvester region performed better than those where force is concentrated further downstream. As illustrated in tests 1 and 5, there appears to be very little difference in performance between the front-loaded and slightly broader distributions, provided there is force applied at the beginning of the harvester region. As test 6 shows, there comes a point where the force delivery is too staggered, resulting in *C*_*p*_ losses.

Noting that front-loading the distribution of the side force appears to be beneficial, we may wonder to what extent this should be pursued. We consider linear distributions of the side force along the streamwise direction

$$\begin{array}{c} C_{y}\left(\frac{x}{L}\right)=\frac{\text{d}C_{y}}{\text{d}x/L}. \end{array}$$ (14)

**Fig 15:**
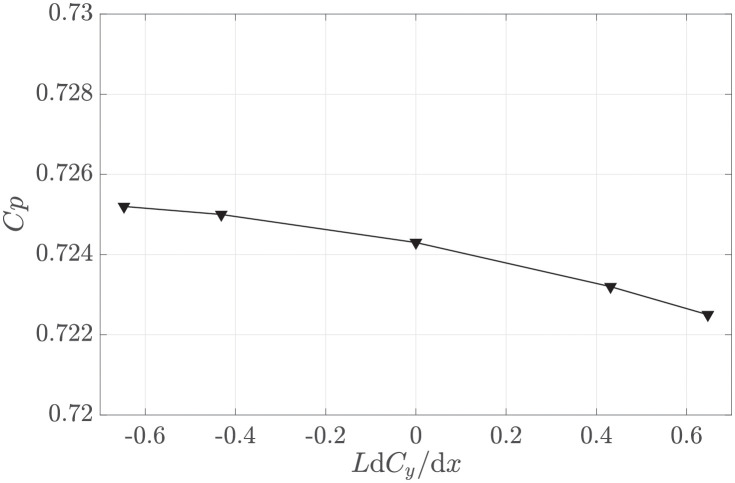
Power coefficient versus the nondimensional slope of the linear streamwise side force distribution. Side force is exerted by deflector type $F_{x_{3}}$ at the side edges of the harvester region. The thickness of the deflector region is *T* = 0.1*W*.

Fig 15 shows *C*_*p*_ for different slopes of the linear distributions. Results are in agreement with Fig 14, demonstrating that an upstream-biased side force distribution (negative gradients) increases the maximum available *C*_*p*_ in the harvester region. A negative gradient yielded better performance, but the trend shows a plateau for highly front-loaded distributions.

## 6 Discussion

The first research question that this paper aims to address is how the harvested power varies by exerting a side force distributed over the array. The curve for $F_{x_{1}}$ in Fig 12 clearly demonstrated that exerting a side force across the whole array is not beneficial. This is because, for each point where a side force is exerted, the harvested power increases on the inner side of the array and decreases on the outer side. The overall balance is mildly negative. For example, for a side force coefficient *C*_*y*_ = 1, the power coefficient decreases by 1%. We therefore conclude that the side force should be exerted from the edges of the array, and not from the inside of the array.

For the same reason, when a device both exerts a side force and harvess power, the part of the device generating side force should be located on the side of the device in closest proximity to the outer edge of the array. Consider, for example, an array made up of devices comprising a turning vane to deflect the flow and a turbine to harvest power. The turning vane should be located on the side of the device nearest to the outer edge of the array, and the turbine should be located on the side of the device nearest to the centre of the array. Each device harvests more power with its own turning vane than without, but the turning vanes of neighbouring devices lowers the power available to the individual device. Overall, the power harvested by the array in this case remains approximately unchanged.

Consider instead an array made up of yawed turbines. Each turbine would both harvest power and deflect the flow outwards. In this case, half of each turbine would harvest more power, while the other half would harvest less power, and the net power increase would be zero for both each individual turbine and for the whole array.

This analysis shows that the side force should be exerted from the edges of the array. Furthermore, if devices that both exert a side force and harvest power are used on the edges of the array, the side force generation should occur on the outer side of the device. Therefore, the region where power is harvested should be entirely inside of the region where side force is exerted. This conclusion addresses the key research questions of the paper: each device within the array should *either* harvest power or exert a side force, depending on its position.

In §5, we investigated the optimal configuration, where deflectors that do not harvest power are placed at the edges of the array. In the following, we estimate whether it would be more convenient to adopt harvesters or deflectors on the side of the array.

Fig 9 shows that the maximum power coefficient only marginally depends on the streamwise length of the array *L*. Therefore, we can equally apply these results to an array made of one or few rows (*L* ≪ *W*) or several rows (*L* ≈ *W*). Consider, for example, an array of *m* rows of *n* side-by-side harvesters spaced by *D* such that *W* = *nD*. At each end of a row of harvesters, we place a deflector. Collectively, the deflectors generate a side force *F*_*y*_ over a length *L*. Assume that each deflector has a dimension *D* equal to the harvester spacing such that *L* = *mD*.

It is noted that, in the case of tidal turbines and for the optimal local blockage *B* = 0.4 [8], the optimal turbine separation *D* is of the same order of magnitude of the turbine diameter. Therefore, the turning vane could be a wing with a chord length of the order of the turbine diameter, and a span ranging from the seabed to the free surface.

The side force per unit volume generated by the vane with lift coefficient *C*_*l*_ is

$$\begin{array}{c} F_{y}=\frac{1}{2}\rho U_{\infty }^{2}W^{-1}C_{l}. \end{array}$$ (15)

Substituting into Eq 5, we find that

$$\begin{array}{c} C_{y}=\frac{U_{\infty }^{2}}{V^{2}}\frac{L}{W}C_{l}. \end{array}$$ (16)

The ratio *U*_∞_/*V* is of the order of *C*_*l*_/*C*_*d*_, and *L*/*W* = *mD*/(*nD*) = *m*/*n*. Therefore, Eq 16 becomes

$$\begin{array}{c} C_{y}\approx \frac{C_{l}^{3}}{C_{d}^{2}}\frac{m}{n}. \end{array}$$ (17)

It is noted that for an array, it is reasonable to scale the dimension of the deflectors with the that of the harvesters, as opposed to that of the array, and consequently the side force coefficient scales with *m*/*n*.

Fig 13 shows that the power increases linearly with the side force, where ∂*C*_*p*_/∂*C*_*y*_ ≈ 0.3 and $C_{p_{0}}\equiv C_{p}\left(C_{y}=0\right)\approx 0.6$. Hence, the relative increase in the power coefficient is

$$\begin{array}{c} \frac{\Delta Cp}{C_{p_{0}}}=\frac{\partial C_{p}}{\partial C_{y}}\frac{Cy}{C_{p_{0}}}\approx \frac{1}{2}\frac{C_{l}^{3}}{C_{d}^{2}}\frac{m}{n}. \end{array}$$ (18)

Finally, we explore whether it would be more convenient to deploy an additional harvester instead of each deflector. In this case, because the power increases linearly with the area spanned by the array, the relative power increase would be 2*D*/*W* = 2*D*/(*nD*) = 2/*n*. By comparison with Eq 18, we find that deploying a deflector at each edge of a row of harvesters would be more beneficial than deploying an additional harvester if

$$\begin{array}{c} m>4\frac{C_{d}^{2}}{C_{l}^{3}}. \end{array}$$ (19)

Even for a single row of harvesters (*m* = 1), it is realistic to design a deflector such that Eq 19 is satisfied. Even if deflectors could not satisfy Eq 19, these could still be more economically viable because cheaper to manufacture and maintain than a harvester.

We conclude that for an array of harvesters in a constrained flow such as in tidal energy, deflectors should be considered at the edge of each row of harvesters.

## 7 Conclusions and future work

In this paper, we investigate the power extraction limit for a two-dimensional harvester region in an infinitely wide channel. This is a simple model of a tidal turbine array, where turbines are closely packed and the array spans across a negligible fraction of the channel width. We consider exerting a side force in the streamnormal direction to oppose the expansion of the streamtube due to the power extraction.

We solve the two-dimensional Reynolds-averaged Navier-Stokes equations, where the power harvesting and the side force are modelled by a source term in the momentum equation. The ability of the model to correctly predict the physical phenomena of interest is verified as follows. In the absence of a side force, the model correctly predicts the maximum power coefficient set by Lanchester-Betz-Joukowsky’s law, with small errors due to the finite domain size and the grid resolution. For a uniform side force exerted within the array, the model predicts a deflection of the streamtube axis that is in good agreement with potential flow theory.

First, we consider the side force to be exerted by the power-harvesting devices within the array. The results show that the side force should not be exerted by the harvesters but by dedicated deflectors deployed at the sides of the array.

Then, we consider a harvester region with a layer of deflectors along each side edge. The deflectors along the edges exert an outward side force, while power extraction occurs within the harvester region. It is shown that the maximum harvested power increases linearly with the side force. The power increases further when the upstream deflectors exert higher force than those downstream. Overall, these results suggest that to maximise the power extraction, future arrays might be equipped with deflectors at the side edges of the array.

An equation (Eq 19) describing the condition when deflectors should be employed is proposed. It shows that deflectors should be employed when they can deliver a high lift to drag ratio, and their effectiveness increases with the number of rows contained in the array.

It is noted that this is a theoretical study with a high level of abstraction, and thus it aims to identify new promising research directions rather than conclusively identify technological solutions. First, future works should consider a more detailed representation of the array, modelling individual harvesters and deflectors. The optimal spacing *D* between harvesters (and thus the optimal local blockage *B*) might depend on the side force and the position of the harvester within the array. Furthermore, it is envisaged that the high shear flow generated by the deflector might pose a challenge to the operation of the harvesters in closest proximity to the deflectors.

Within this work we consider the power extracted from the flow, as in Lanchester-Betz-Joukowsky’s theory. We do not consider the power extraction efficiency, viz. the ratio between the power delivered to the electrical grid and that extracted from the flow. This efficiency depends on the specific technology. Hence, both the maximum power and the potential gains due to the deflectors are to be considered as upper limits and exact values should be assessed in future works.
